# Meropenem-induced pancytopenia in a preterm neonate: a case report

**DOI:** 10.1186/s13256-020-02632-1

**Published:** 2021-01-29

**Authors:** Kashif Hussain, Muhammad Sohail Salat, Naureen Mohammad, Ambreen Mughal, Sidra Idrees, Javaid Iqbal, Gul Ambreen

**Affiliations:** 1 Department of Pharmacy, Main Pharmacy Aga Khan University Hospital, Stadium Road, P.O Box 3500, Karachi, 74800 Pakistan; 2 Department of Paediatrics & Child Health, Aga Khan University, Karachi, Pakistan

**Keywords:** Meropenem, Preterm neonate, Pancytopenia, Absolute neutrophil count, Hematological problems

## Abstract

**Background:**

A post-marketing surveillance study has reported an association between meropenem use and the incidence of hematologic abnormalities, including leukopenia, thrombocytopenia, hemolysis, and neutropenia, but the precise incidence in neonates is unknown. Here, we report meropenem-induced pancytopenia in a preterm neonate.

**Case presentation:**

A preterm newborn Pakistani received intravenous meropenem 40 mg/kg every 8 hours to treat *Klebsiella pneumoniae* in blood cultures and suspected meningitis. The baby developed severe thrombocytopenia, with a platelet count of 22 × 10^3^ cells/mm^3^, low hemoglobin level of 9.7 g/dl, and low absolute neutrophil count (ANC) of 816 cells/mm^3^ on days 3, 14, and 17 of meropenem therapy, respectively. Based on the blood culture and institutional guidelines, meropenem treatment was continued with monitoring and supportive care for a total of 19 days. After discontinuation of meropenem, the baby was monitored continuously for hematological changes, and low counts persisted for 3 days. ANC improved to > 1500 cells/mm^3^ on the fourth day, and the platelet count reached > 150 × 10^3^ cells/mm^3^ for the first time on the seventh day of meropenem discontinuation. All subsequent complete blood count (CBC) reports showed improving trends. The baby was discharged on the 48th day of life (DOL), with follow-up monitoring of CBC. The baby was kept on iron supplements, and hemoglobin level of 11.2 g/dl was observed on the 59th DOL.

**Conclusion:**

Neonatal pancytopenia may lead to serious health complications; therefore, clinicians and pharmacists need to vigilantly monitor CBC in this vulnerable population, even when administering meropenem in septic doses for the recommended duration.

## Background

Pancytopenia is defined as reduced white blood cell (WBC) count, hemoglobin, and platelet count. Pancytopenia occurs when hemoglobin is < 13 g/dl in males and < 12 g/dl in females, absolute neutrophil count (ANC) is < 1500 cells/mm^3^, and platelet count is < 150 × 10^3^ cells/mm^3^ [1]. Pancytopenia is considered severe if a patient experiences two or more of the following: hemoglobin < 7 g/dl, ANC < 500 cells/mm^3^, and platelet count < 50 × 10^3^ cells/mm^3^. The mechanism underlying pancytopenia mainly involves bone marrow infiltration, bone marrow aplasia, and blood cell destruction that results in peripheral blood leakage [1]. Suppression of bone marrow varies widely in the pediatric population but may occur due to toxins, infection, or malignant cell infiltrates that can lead to hypocellular bone marrow function. Drug-induced pancytopenia is also a rare but secondary cause of bone marrow suppression due to direct bone marrow toxicity, immune-mediated (complement or antibody-mediated) cell destruction, and hapten formation, and directly affects myeloid precursors [2, 3]. An *in vitro* study of several beta-lactam antibiotics established the presence of well-differentiated myeloid cells and copious granulocyte precursors along with dose-dependent suppression of granulopoiesis [4]. Antibody-mediated hemolytic anemia and thrombocytopenia have also been established in meropenem-treated patients [5, 6]. Several medications can cause pancytopenia including chemotherapeutics, antiepileptics, antidepressants, and antibiotics [3, 5, 7]. A case–control epidemiological surveillance study was conducted over a follow-up period of 78.7 million person-years to assess the incidence of drug-induced agranulocytosis. Around 396 confirmed cases of acute neutropenia were observed, with an overall incidence of 3.5:1 million per year [8]. It was found that agranulocytosis clearly increased the risk of mortality, with a fatality rate of 9.1%. The most common drugs causing agranulocytosis were dipyrone (16%), beta-lactam antibiotics (12.0 %), ticlopidine (11.1%), antithyroid drugs (7.2%), and sulfonamide antibiotics (5.4%) [8].

Meropenem is one of the beta-lactam antibiotics that can cause serious and life-threatening neutropenia. It is a bactericidal broad-spectrum antibiotic with gram-positive, gram-negative, and anaerobic coverage, and is mainly used in the treatment of severe gram-negative infections in neonates including severe sepsis, meningitis, and complicated intra-abdominal infections such as necrotizing enterocolitis (NEC), one of the most common gastrointestinal emergencies and a major cause of morbidity and mortality in preterm neonates. For improved clinical outcomes, early recognition and aggressive management with broad-spectrum or combination antimicrobial agents is most often undertaken to treat NEC [9]. Because of its broad-spectrum activity, meropenem is an agent of great utility [10]. Based on the increasing trends of morbidity and mortality due to multidrug-resistant gram-negative bacterial infections in our neonatal intensive care unit (NICU), and following institutional guidelines, other antibiotics such as vancomycin and colistin are also used [11–13] after infectious disease (ID) consultation. The most common adverse effects of meropenem are constipation or diarrhea, nausea, vomiting, rashes, and diaper-area moniliasis in pediatric patients [14]. Some cases of meropenem-induced neutropenia have been reported [15]. However, no known case of meropenem-induced pancytopenia in neonates has yet been published. Here we report an event of meropenem-induced pancytopenia in a neonate admitted to the NICU of a tertiary-care hospital.

## Case presentation

A newborn Pakistani baby was transferred to the intensive care unit due to prematurity, low birth weight, and intrauterine growth retardation. Her birth weight was 0.71 kg and APGAR scores were 3 at 1 minute and 4 at 5 minutes. Delivered by emergency C-section at 29 weeks due to raised blood pressure and fetal distress, she was initially kept on continuous positive airway pressure (CPAP) and given nothing through the mouth, and total parenteral nutrition (TPN) was started. Caffeine was loaded at 20 mg/kg and continued as the standard of care for apnea of prematurity. A prophylactic dose of 1 mg of vitamin K was given. During the first 24 hours, complete blood count (CBC) results were WBC of 18.3 × 10^3^ cells/mm^3^, ANC of 1244 cells/mm^3^, platelet count of 166 × 10^3^ cells/mm^3^, and hemoglobin of 17.8 g/dl. On the second day of life (DOL), ampicillin and gentamicin were started as empirical therapy, and the CBC report showed WBC 4 × 10^3^ cells/mm^3^, ANC of 760 cells/mm^3^, platelet count of 152 × 10^3^ cells/mm^3^, and hemoglobin of 15.9 g/dl. In addition, fluconazole was started as antifungal prophylaxis. The patient was given phototherapy. Chest X-ray and ultrasound of the head were performed, with normal findings. CPAP was tapered to high flow on merit, and blood culture was sent.

On the fourth DOL, the baby developed issues of severe respiratory distress and abdominal distension along with metabolic acidosis. She was intubated and kept on synchronized intermittent mandatory ventilation (SIMV) mode. The CBC results showed ANC of 912 cells/mm^3^, platelet count of 99 × 10^3^ cells/mm^3^, and hemoglobin of 12.7 g/dl. Inotropic support was started, and antibiotics were escalated to meropenem 20 mg/kg every 12 hours, vancomycin 10 mg/kg once daily, and colistin at a loading dose of 5 mg/kg with a maintenance dose of 1.5 mg/kg every 12 hours. Blood culture showed no growth. After ID consult, vancomycin was discontinued on the third day and a decision was made to continue meropenem and colistin [11–13] to manage NEC and sepsis.

On the fifth DOL, echocardiogram was performed and showed patent ductus arteriosus (PDA) of 3 mm with severe persistent pulmonary hypertension of the newborn (PPHN). Acetaminophen was started for the next 5 days. On the sixth day of meropenem therapy, the platelet count dropped to 42 × 10^3^ cells/mm^3^, treated in line with sepsis-associated thrombocytopenia [16], and managed by transfusion of 10 ml/kg platelet units [17]. Meropenem and colistin were discontinued on the eighth day of therapy, and trophic feeding was started. An echocardiogram was repeated on day 11, which showed a closed PDA and moderate PPHN. On the 18th DOL, the child was successfully extubated and kept on high-flow oxygen. There was slow progress in feeding.

On the 22nd DOL, the baby had tachycardia and an episode of 99.7 °F fever. Thus, septic workup was done and showed WBC count of 17.4 × 10^3^ cells/mm^3^, ANC of 12,632.4 cells/mm^3^, and platelets of 203 × 10^3^ cells/mm^3^. C-reactive protein (CRP) was 77 mg/l and renal function was normal (blood urea nitrogen [BUN] of 11 mg/dl and creatinine of 0.2 mg/dl). Meropenem was started in meningitic doses of 40 mg/kg every 8 hours [10] along with vancomycin 15 mg/kg every 12 hours.

In the microbiological investigation, urine and cerebrospinal fluid (CSF) cultures were found to be negative, but the blood culture was positive for *Staphylococcus* species (not *aureus*). Blood culture repeated after 48 hours was positive for carbapenem-sensitive *Klebsiella pneumoniae*. The baby was moved to the isolation room. On the 24th DOL, the baby developed severe thrombocytopenia, with a platelet count of 22 × 10^3^ cells/mm^3^, managed with the transfusion of 10 ml/kg platelet units [17].

On the 25th DOL, an episode of generalized tonic-clonic fit occurred for which single-dose diazepam and a loading dose of phenobarbitone were given initially and then continued with maintenance doses of phenobarbitone. Due to significant metabolic acidosis and desaturation, the baby was re-intubated. After ID consult, meropenem was continued in meningitic doses and vancomycin was discontinued. SIMV and a central line were placed. TPN was started with nothing per oral status.

Blood gases were monitored, and the baby was extubated after 4 days and switched to CPAP. Phenobarbitone was discontinued after 6 days of therapy, and the baby was seizure-free through the remainder of the hospital stay. All repeat blood cultures were negative for any growth including CSF culture. The ID team was on board and decided to continue meropenem for a total of 14 days post-negative culture in septic doses of 20 mg/kg, and vancomycin was discontinued after 6 days. All repeated blood cultures obtained on the 27th and 30th DOL confirmed no pathogenic growth.

On the 33rd DOL, the CBC report showed WBC of 8.7 × 10^3^ cells/mm^3^, ANC of 4576 cells/mm^3^, platelets of 78 × 10^3^ cells/mm^3^, and hemoglobin of 10.8 g/dl. On the 36th DOL, the CBC report showed WBC of 5.1 × 10^3^ cells/mm^3^, ANC of 2917 cells/mm^3^, platelets of 29 × 10^3^ cells/mm^3,^ and hemoglobin of 9.2 g/dl. The baby was managed for thrombocytopenia with platelet transfusion.

On the 37th DOL, hemoglobin dropped to 8.3 g/dl in the evening, and platelet count improved to 63 × 10^3^ cells/mm^3^, WBC was 4.8 × 10^3^ cells/mm^3^, and ANC was 1285 cells/mm^3^. On the 39th DOL, a marked reduction was observed in ANC to 816 cells/mm^3^, platelets of 38 × 10^3^ cells/mm^3^, and hemoglobin of 8.3 g/dl, and the baby received blood transfusion to manage it. For constant issues, a hematological consult was taken and a decision was made to manage the baby symptomatically with 10 ml/kg platelet units at a platelet count < 50 [17] and 15 ml/kg packed cell transfusion at hemoglobin < 10 g/dl [18].

**Fig. 1.**
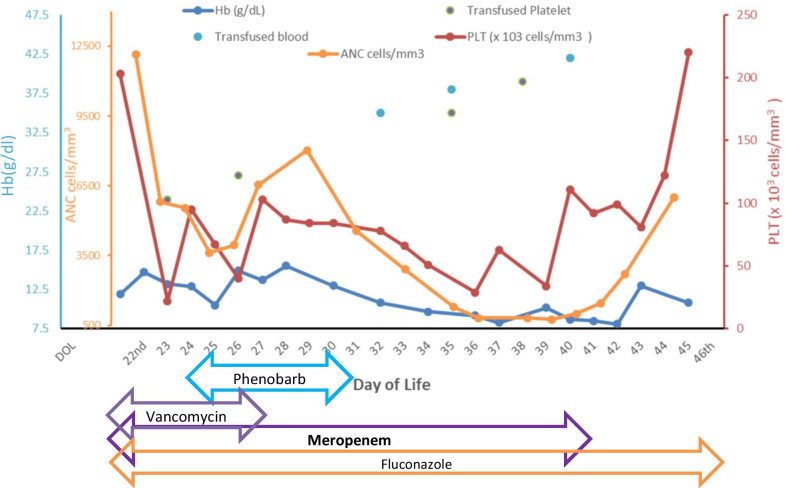
Hematological changes and timing of medication during hospitalization

On the 40th DOL, meropenem was discontinued 14 days after negative culture (total of 19 days of meropenem therapy). On the same day the platelet count was 34 × 10^3^ cells/mm^3^, ANC was 818 cells/mm^3^, and hemoglobin was 10.2 g/dl, and the baby was transfused to manage these issues. After discontinuation of meropenem, the baby was continuously monitored for hematological changes, and low counts persisted for 3 days. Improved ANC of > 1500 cells/mm^3^ was reported on the fourth day, and platelet count of > 150 × 10^3^ cells/mm^3^ was reported for the first time on the sixth day of meropenem discontinuation, but hemoglobin was still low (Fig 1). CPAP was tapered gradually to nasal prongs. The peripherally inserted central catheter was removed, and orogastric (OG) tube feeding was commenced. The baby was discharged on the 48th DOL on iron supplements with follow-up monitoring of CBC. Hemoglobin level of 11.2 g/dl was found on the 59th DOL.

## Discussion

Drug-induced pancytopenia is a rare hematological problem in neonatal clinical practices, evaluated based on a complete underlying pathological history, physical examination, and vigilant interpretation of biochemical, radiological, histopathological, and hematological findings [19]. Concerns have been raised about hematological adverse effects of several beta-lactamase inhibitors [8]. This neonatal case documents meropenem-induced pancytopenia and emphasizes cautious laboratory monitoring for patients receiving meropenem therapy.

Sepsis is another known etiology of pancytopenia. Although the neonate in the present case had a blood culture positive for *K. pneumoniae*, the results of all repeated blood cultures were negative, showing the resolution of sepsis. Van Tuyl *et al.* [20] also reported a case of a neonate receiving meropenem in meningitic doses of 40 mg/kg/day to treat meropenem-susceptible *Enterobacter cloacae* in blood culture. The baby developed meropenem-induced neutropenia on the 13th day of therapy, with ANC of 288 cells/mm^3^. The decision was made to discontinue meropenem on the 19th day of therapy instead of the initially planned 21 days. In the present case, the baby continued to receive meropenem treatment and was managed for hematological problems based on institutional guidelines and culture sensitivity. Initially, hematological changes were not considered as directly meropenem-induced.

Recently published reports also support the notion of carbapenem-induced hematological disorders. A case report by Estella and colleagues [21] described meropenem-induced pancytopenia in a 3-year-old patient at 100 mg/kg given every 8 hours to manage the regrowth of meropenem-sensitive *Morganella morganii* in CSF cultures. Huang *et al.* [6] reported a case of meropenem-induced immune thrombocytopenia in a 59-year-old patient, by detecting meropenem-dependent platelet antibodies and platelet count recovery after discontinuation of meropenem. Oka *et al.* [5] reported the development of severe anemia with clinical signs in a 76-year-old female patient, who received 2 g meropenem per day. On further investigation, they found that a direct antiglobulin test (DAT) was positive for immunoglobulin G (IgG) and C3d, and reported the presence of meropenem-dependent antibodies in the patient’s serum [5].

The absence of drug-dependent antibody testing and bone marrow aspiration prevent us from drawing a firm conclusion regarding the mechanism of meropenem-induced pancytopenia in the present case. However, the hematological findings with the progressive development of thrombocytopenia, anemia, and then neutropenia suggest a possible mechanism of suppression of granulopoiesis or antibody-mediated destruction, as reported in recent studies [5, 6].

During hospitalization, the neonate received other medications, which have been reported to exert hematological effects. The baby initially received ampicillin, gentamicin, and fluconazole, and then received meropenem, colistin, vancomycin, and phenobarbital. However, severe thrombocytopenia developed on the sixth day of the first course of meropenem therapy, when the baby was also on colistin and fluconazole. This issue was resolved when meropenem was discontinued. During the second course of meropenem therapy, the baby again developed severe thrombocytopenia on the third day, while also receiving vancomycin, fluconazole, and phenobarbital. Phenobarbital-induced hematological abnormalities are reported in animal and adult studies [22, 23], and therefore it was discontinued on the sixth day. No seizures were observed but counts did not improve. However, the baby developed severe anemia and neutropenia while receiving fluconazole and meropenem only. After meropenem discontinuation, severe anemia and neutropenia showed a resolving trend for 3 days. Severe neutropenia resolved on the fourth day, and hemoglobin was reported at > 10 g/dl without transfusion on the seventh day of meropenem discontinuation, while fluconazole therapy was continued from the beginning until the 56th DOL, which further confirms the association of meropenem-induced hematological changes in this baby. It was also confirmed that the baby did not experience any additional infection even though she was not discharged on any antibiotic, which correlates with the fast recovery of blood counts after meropenem discontinuation.

## Conclusions

In the present neonatal case, the gradual onset of hematological irregularities correlates with the commencement of meropenem therapy, and our observations are also verified by previously published evidence. The follow-up CBC count further established the resolution of pancytopenia after discontinuation of meropenem. Neonatal pancytopenia may lead to serious health complications; therefore, clinicians and pharmacists need to vigilantly monitor CBC counts in this vulnerable population, even when administering meropenem in septic doses for the recommended duration.

### Patient perspective

From the perspective of the patient’s father, it was a rare case to have such kind of adverse events in a neonate with the normal treatment regimen. He was shared about all the events and he admired the team for the timely and effectively managing the issues and finally 100% recovery on the follow-ups.

## Data Availability

All data generated or analyzed during this study are included in the published article. The data sets used and/or analyzed during the current study are available from the corresponding author on reasonable request. Please contact the author for data requests.

## Abbreviations

ANC: Absolute neutrophil count
CBC: Complete blood count
DOL: Day of life
CPAP: Continuous positive airway pressure
TPN: Total parenteral nutrition
SIMV: Synchronized intermittent mandatory ventilation
NEC: Necrotizing enterocolitis
PDA: Patent ductus arteriosus
PPHN: Persistent pulmonary hypertension of the newborn
CRP: C-reactive protein
ID: Infectious disease
